# Circ_0058063 contributes to cisplatin-resistance of bladder cancer cells by upregulating B2M through acting as RNA sponges for miR-335-5p

**DOI:** 10.1186/s12885-022-09419-1

**Published:** 2022-03-23

**Authors:** Ming Sun, Xuefeng Liu, Wenyan Zhao, Bin Zhang, Peng Deng

**Affiliations:** 1 Department of Urology, Shengjing Hospital of China Medical University, NO. 36 Sanhao Street, Heping District, Shenyang City, 110004 Liaoning Province Shenyang China; 2 Department of General Surgery, Shengjing Hospital of China Medical University, Shenyang, 110004 China; 3 Department of Surgical Oncology and General Surgery, Key Laboratory of Precision Diagnosis and Treatment of Gastrointestinal Tumors, Ministry of Education, The First Affiliated Hospital of China Medical University, Shenyang, 110001 China

**Keywords:** Bladder cancer, Cisplatin resistance, circ_0058063, miR-335-5p, B2M

## Abstract

Bladder cancer (BC) is one of the most common malignant tumors of the urinary system, and cisplatin (CDDP) is a critical chemical drug for the treatment of BC. However, CDDP-resistance seriously limits the therapeutic efficacy of this drug for clinical utilization. Thus, identification of pivotal molecule targets that regulate CDDP-resistance in BC become urgent and necessary. In this study, we firstly identified a novel BC-associated circular RNA circ_0058063 that participates in the regulation of CDDP-resistance in BC. Specifically, circ_0058063 was significantly overexpressed in CDDP-resistant tissue and cells, in contrast with the corresponding CDDP-sensitive counterparts. Further loss-of-function experiments validated that downregulation of circ_0058063 suppressed cell proliferation and tumor growth, whereas induced cell apoptosis in the CDDP-resistant BC cells *in vitro* and *in vivo*. In addition, we disclosed that circ_0058063 acts as a sponge for miR-335-5p to positively regulate B2M expression, and further rescuing experiments verified that the enhancing effects of sh-circ_0058063 on CDDP-sensitivity in the CDDP-resistant BC cells were abrogated by silencing miR-335-5p. Taken together, our results demonstrated that circ_0058063 contributed to CDDP resistance of bladder cancer cells via sponging miR-335-5p, and B2M might be the downstream effector gene. This study firstly evidenced that targeting circ_0058063 might be an effective strategy to improve CDDP-sensitivity in BC.

**Supplementary Information:**

The online version contains supplementary material available at 10.1186/s12885-022-09419-1.

## Introduction

Bladder cancer (BC) is the most common malignant tumor of the urinary system that occurs on the bladder mucosa. Currently, BC treatment methods mainly include surgical resection, additional local or systemic immunotherapy, chemotherapy and radiotherapy [1, 2]. However, the resistance of BC to conventional therapies (such as radiotherapy, chemotherapy and immunotherapy) is the main cause of BC recurrence and worse prognosis [3]. Cisplatin (CDDP) is one of the commonly used chemical drug in BC chemotherapy [4]. Nevertheless, drug resistance to CDDP seriously limits the therapeutic efficacy of this chemical drug for the treatment of BC. Also, as the results of its unclear molecular mechanisms, there are still no effective adjuvant therapy strategies to improve CDDP-sensitivity. Therefore, it is very important to clarify the molecular mechanism involved in the CDDP chemoresistance of BC cells, which is beneficial to the development of effective chemotherapeutics treatment of BC.

Circular RNAs (circRNAs) are a kind of non-coding RNAs with circular structure [5]. CircRNAs have been widely revealed to play an important role in various human diseases, including cancers, cardiovascular disease [6], renal disease [5], and heart disease [7]. For instance, has_circ_100395 suppressed lung cancer progression via miR-1228/TCF21 pathway [8]. Circ_BPTF was examined to boost cell proliferative, migratory and invasive through the miR-31-5p/RAB27A axis in bladder cancer [9], and exosomes-containing circ_0044516 increased cell proliferation and metastasis in human prostate cancer [10]. Moreover, circRNAs also regulate chemo-resistance in cancers. Specifically, circ_0071589 was reported to enhance CDDP resistance in colorectal cancer [11], and circ_0000260 induced CDDP-chemoresistance in gastric cancer [12]. As one of the novel circRNAs, circ_0058063 is recently identified as an oncogene to facilitate cancer aggressiveness in multiple myeloma [13], bladder cancer [14, 15], and promoted glucose-uptake in esophageal squamous cell carcinoma [16], but it is still unclear whether circ_0058063 is involved in modulating CDDP-resistance in BC.

The most classic mechanism of circRNA is to compete for endogenous RNA (ceRNA). circRNAs exert their biological functions by acting as RNA sponges of microRNAs (miRNAs), and have been widely reported in various types of cancer [17]. For ovarian cancer, circCELSR1 was found to contributes to paclitaxel resistance via regulated FOXR2 by sponging miR-1252 [18]. Also, Huang et al. uncovered that the circular RNA AKT3 sponge miR-198 improved PIK3R1 expression to enhance CDDP resistance in gastric cancer [19]. In our previous research, we have demonstrated than Circ_0058063 promote bladder cancer progression by sponging miR‐145‐5p [20]. Nonetheless, the function of circ_0058063 as miRNA sponges has not been clearly disclosed in BC resistance to CDDP. Among all the miRNAs, miR-335-5p is reported to act as a tumor suppressor to suppress cancer malignancy in lung adenocarcinoma [21], osteosarcoma [22], colorectal cancer [23], and regulate CDDP-resistance in ovarian cancer [24]. Interestingly, our bioinformatics analysis revealed that there existed potential targeting relationship between circ_0058063 and miR-335-5p.

Collectively, in this study, we explored the function of circ_0058063 and the mechanism in CDDP-resistant BC tissues and cells. Firstly, we found that the expression of circ_0058063 is markedly improved in CDDP-resistant BC tissues and cell lines. We further indicated that circ_0058063 upregulate the level of B2M by sponging miR-335-5p and contribute CDDP-resistant of BC cells by promoting the expression of genes related to the properties of cancer stem cells. Furthermore, miR-335-5p inhibitor reverses the inhibition of cell proliferation and the promotion of apoptosis by silencing circ_0058063. Our findings will provide new ideas for the regulation mechanism of circ_0058063 in BC progression and CDDP resistance.

## Materials and methods

### Patients and samples

Bladder cancer (BC) specimens were collected from bladder cancer patients receiving cystectomies between August 2019 and February 2020 at the Department of Urology, Shengjing Hospital of China Medical University. Subsequently, the BC patients were divided into the CDDP-resistant group (*n* = 16) and the CDDP-sensitive group (*n* = 19), according to standard CDDP response published elsewhere [25]. CDDP-resistance was defined as tumor recurrence during CDDP-based chemotherapy after R0 resection, and CDDP-sensitivity was defined as the absence of tumor recurrence during CDDP-based therapy. This study only collected samples of BC patients, and did not involve gender differences. The study did not conduct gender analysis. The samples were stored in the refrigerator at -80℃ until use. This study was conducted in accordance with the “Declaration of Helsinki” and was approved by the Ethics Committee of Shengjing Hospital Affiliated to China Medical University, and written informed consent was provided by each patient.

### Cell culture and transfection

Human BC cell lines (T24 and 5637) and a normal human urothelial cell line (SV-HUC-1) were purchased from American type culture collection (ATCC, Beijing, China). The corresponding CDDP-resistant BC cells T24/CDDP and 5637/CDDP cells were established from the T24 and 5637 parental cell lines by stepwise exposure to increasing CDDP concentrations, as previously described [26]. All cells were cultured in Dulbecco’s modified Eagle’s medium (DMEM) supplemented with 10% fetal bovine serum (FBS) in a 95% air and 5% CO2 atmosphere at 37 ℃. In addition, the downregulation vectors of circ_0058063, and miR-335-5p mimic and inhibitor were constructed by Guangzhou RiboBio Co., Ltd. (Guangzhou, China) according to the previous publications [15, 22, 27].

### RNA extraction, treatment with RNase R, and PCR

RNA extraction of BC tissues and cells was using TRIzol reagent (Invitrogen). For RNase R treatment, 2 μg total RNA was incubated for 1 h at 37 °C with or without 3 U/μg of RNase R (Epicentre Technologies, Madison, WI, USA). After treatment with RNase R, real-time quantitative PCR (RT-qPCR) was performed to determine the expression of cicr_0058063.

### Fluorescence in situ hybridization analysis (FISH)

The RNA fluorescence in situ hybridization (FISH) probe targeting circ_0058063 was designed and produced by Gene seed Biotechnology Co., Ltd. (Guangzhou, China). T24 and 5637 cells were cultured on coverslips and fixed with 4% paraformaldehyde in PBS for 15 min. FISH probes were diluted, denatured, equilibrated and added to cells overnight at 37 °C. After hybridization and washing, slides were dehydrated and then mounted using Prolong Gold Antifade Reagent and DAPI for further detection. Finally, the results were observed with a fluorescence microscope (DMI4000B, Leica).

### CCK-8 assay

BC cell viability and IC50 value were assessed using the CCK-8 (Yeasen, Shanghai, China). Cells were collected and seeded into 96-well plates at a density of 2 × 10^4^ cells per well for 24 h at 37 °C. Then, 10 μl of CCK-8 solution was supplemented into each well at the indicated time and incubated for 10 min at 37 °C. Absorbance was evaluated at 450 nm through a Rayto-6000 system (Rayto, China).

### Flow cytometry (FCM)

For the apoptosis assay, T24 and 5637 cells were treated with CDDP at the indicated concentrations for 24 h in 6-well plates, they were harvested and resuspended in 300 ml of binding buffer. Next, 5 μl of Annexin V-FITC and 5 μl of PI were added to the suspensions, and the cells were incubated in the dark at room temperature for 15 min. Cell apoptosis was detected using a PI/Annexin V-FITC Apoptosis Detection Kit (BD Biosciences, San Deigo, CA) according to the manufacturer’s instructions.

### Dual luciferase reporter assay

The fragments of circ_0058063 and B2M containing wild type (wt) and mutant (mut) miR-335-5p binding site were sub-cloned into a pmirGLO Dual-luciferase miRNA Target Expression Vector (Madison, WI). Lipofectamine 2000 (Invitrogen) was used to transfect T24 cells and 5637 cells according to the manufacturer’s guidance. MiR‐NC and miR‐335‐5p mimics were co-transfected with pmirGLO empty vector, pmirGLO‐circ‐wt, and pmirGLO‐circ‐mut, pmirGLO‐B2M‐wt, and pmirGLO‐B2M‐mut, respectively. After 48 h of transfection, T24 and 5637 cells were subjected for luciferase activity detection using Luciferase Reporter Assay System (Promega, WI, USA).

### RNA immunoprecipitation (RIP)

T24 and 5637 cells were collected and lysed in the RIP buffer and then mechanically separated utilizing the homogenizer. The cell lysates were added with antibodies against Ago2 at 4 °C overnight. After 24 h, the RNA/bead complex was washed and resuspended in buffer with RNase-free DNase and proteinase K. Finally, RNA was extracted and subjected to RT-qPCR analysis.

### Western blot

Total cell proteins were separated by 12% SDS/PAGE and then transferred to PVDF membrane. The blotted membrane was then blocked with 5% slim milk for 1 h at room temperature and incubated overnight at 4 °C with primary antibodies as follows: B2M, SOX2, OCT4, NANOG and GAPDH. Then, the membrane was incubated with the secondary antibodies for 1 h at room temperature. Protein bands were detected using the enhanced chemiluminescence Western blot analysis Kit (Pierce Chemical, Rockford, IL).

### Nude mouse xenograft model

Six-week-old male BALB/c nude mice were purchased from Shanghai SLAC Experimental Animal Center (China). Mice were subcutaneously injected into the back with 1 × 10^6^ T24/CDDP cells stably transfected with sh-circ_0058063 or sh-NC suspended in 100 μL of Hank’s balanced salt solution. These male nude mice divided into four groups equally (group 1, sh-NC-transfected cells + PBS; group 2, sh-NC-transfected cells + CDDP; group 3, sh-circ-0058063-transfected cells + PBS; group 4, sh-circ-0058063-transfected cells + CDDP). The tumor volume was measured every week according to the formula: volume = (length × width^2^)/2. Animal studies were performed in compliance with the ARRIVE guidelines and the Basel Declaration, and were approved by the Animal Care and Use Committee of Shengjing Hospital Affiliated to China Medical University. All animals received humane care according to the National Institutes of Health (USA) guidelines.

### Immunohistochemical staining (IHC)

Xenografts and gastric cancer tissues were prepared for KI67 immunohistochemical staining as previously described after being treated or injected with the indicated concentrations of cisplatin. Sections were viewed by IHC Imager (DM4000B, LEIKA, Germany).

### Statistical analysis

GraphPad Prism 8.0 was applied to statistical analysis. All data were at least three independent experiments. The statistical analysis of the results was calculated by the t-test and one-way ANOVA. *P* < 0.05 was considered significant.

## Results

### Circ_0058063 was upregulated in CDDP-resistant BC tissues and cells and associated with poor prognosis

**Fig. 1:**
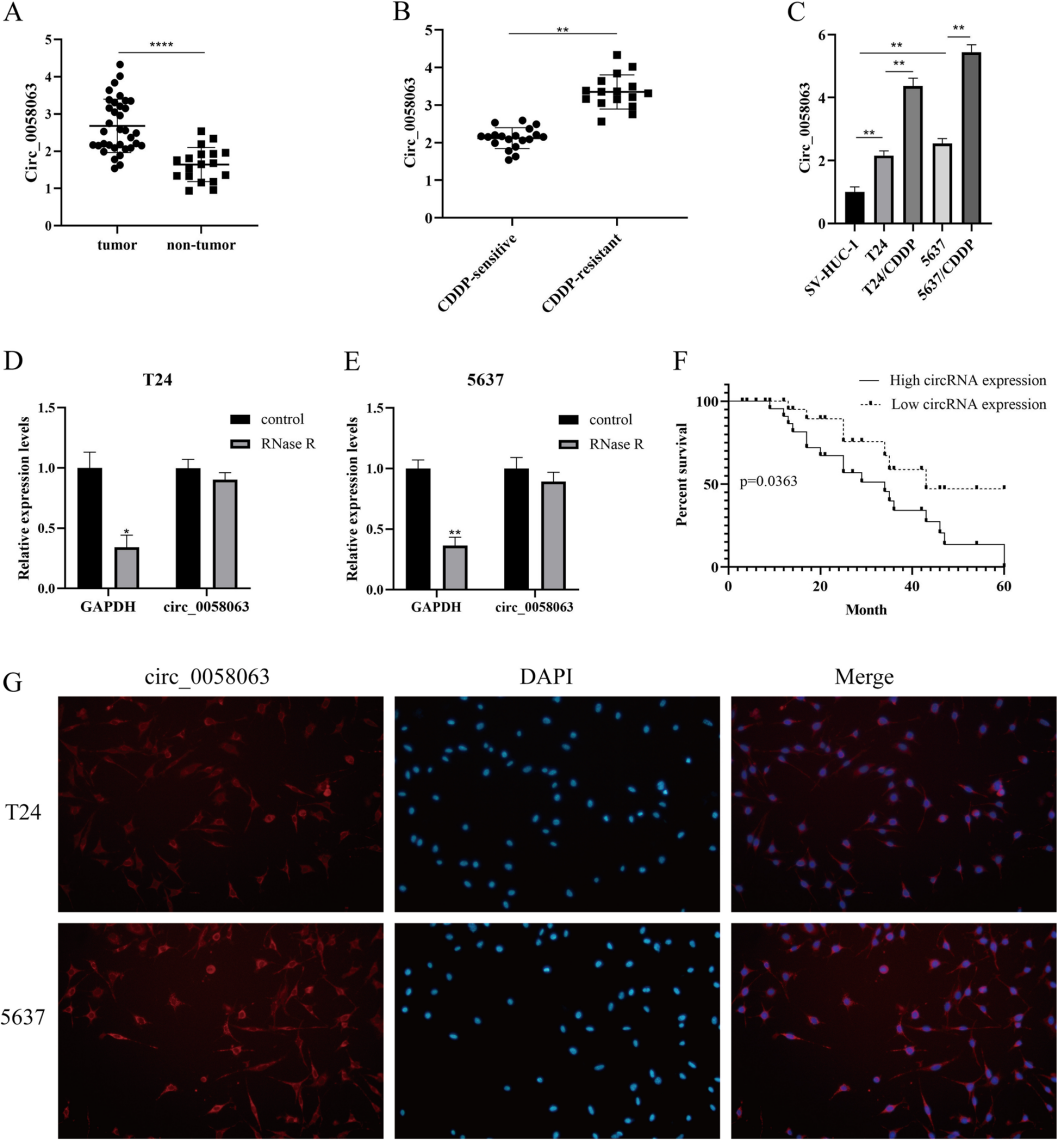
Circ_0058063 was upregulated in CDDP-resistant BC tissues and cells and associated with poor prognosis. **A** Relative expression of circ_0058063 in BC tissue samples (*n* = 35) and nontumorous tissues (*n* = 18). **B** Relative expression of circ_0058063 in CDDP-resistant (*n* = 19) and sensitive BC tissues (*n* = 16). **C** Relative expression of circ_0058063 in SV-HVC-1, T24, T24/CDDP, 5637 and 5637/CDDP cell lines. **D**, **E** The result of RT‐qPCR showed RNase R could not lyse circ_0058063 in T24 and 5637 cells. **F** Kaplan–Meier survival curves of overall survival in BC patients with relatively low or high circ_0058063 expression (*n* = 56). **G** The location of circ_0058063 in T24 and 5637 cells were detected by FISH assay. *P < 0.05

To analyze the expression levels of circ_0058063 in bladder cancer (BC), we first identified the expression of circ_0058063 in BC by RT-qPCR. Circ_0058063 was significantly upregulated in bladder cancer tissues compared with nontumorous (Fig. 1A). Then, RT-qPCR was performed to evaluated the expression of circ_0058063 in CDDP-resistant and CDDP-sensitive BC tissues. As shown in Fig. 1B, a significant upregulation of circ_0058063 in CDDP-resistant BC tissues than in CDDP-sensitive tissues. Moreover, we found that circ_0058063 in CDDP-resistant BC cell lines (T24/CDDP and 5637/CDDP) was higher than that in primary BC cell lines (T24 and 5637) (Fig. 1C). To confirm that circ_0058063 was a circular RNA, we used RNase to digest linear RNA in T24 and 5637 cells. As expected, circ_0058063 was more resistant to RNase treatment in contrast with the linear GAPDH mRNA (Fig. 1D and E). In addition, the overall survival rate of BC patients with high expression of circ_0058063 was lower than that of BC patients with low expression of circ_0058063 (Fig. 1F). Moreover, the fluorescence in situ hybridization (FISH) assay showed that circ_0058063 predominately localized in the cytoplasm of T24 and 5637 cells (Fig. 1G). These resulted suggested that circ-0058063 was increased in CDDP-resistant BC and associated with a poor prognosis.

### Knockdown of Circ_0058063 rescued cell viability and induced apoptosis in CDDP-resistant BC cells

**Fig. 2:**
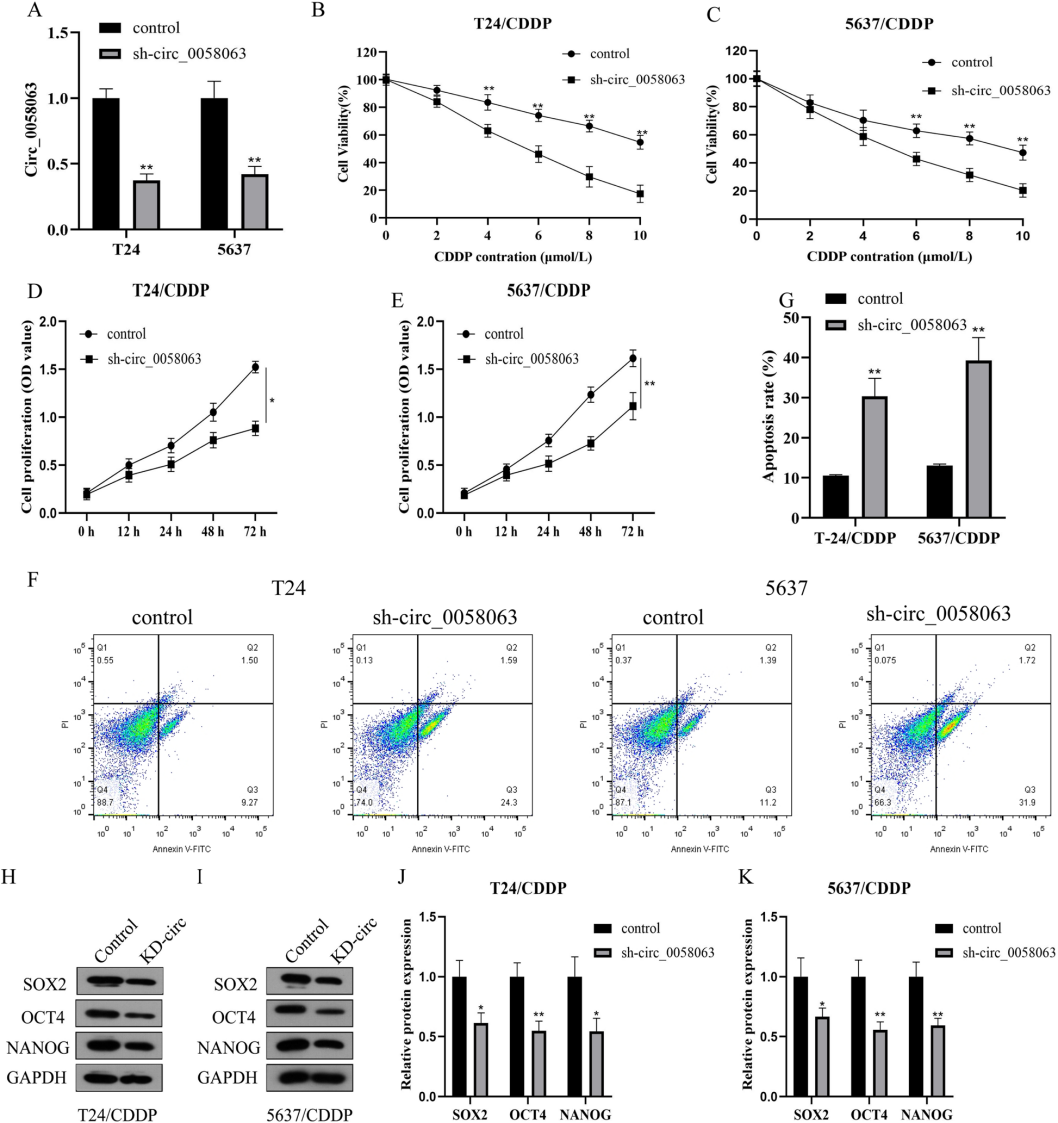
Knockdown of Circ_0058063 rescued cell viability and induced apoptosis in CDDP-resistant BC cells. **A** The transfection efficiency of the circ_0058063 downregulation vectors were examined by RT-qPCR. **B**, **C** The IC50 value of CDDP in T24/CDDP and 5637/CDDP cells transfected with sh-circ_00058063 or sh-NC was assessed by CCK-8 assay for 48 h. **D**, **E** CCK-8 analysis of cell viability of T24/CDDP and 5637/CDDP cells transfected with sh-circ_00058063 or sh-NC. **F**, **G** The apoptosis rate of T24/CDDP and 5637/CDDP cells were examined by FCM assay for 48 h. **H**–**K** The protein levels of SOX2, OCT4 and NANOG in T24/CDDP and 5637/CDDP cells were detected by Western blot. *P < 0.05

To explore the functions of circ_0058063 in BC, the vectors constructed with sh-circ-0058063 or sh-control were injected into T24/CDDP and 5637/CDDP cells, and then the relative expression level of circ_0058063 was detected by RT-qPCR. Sh-circ_0058063 successfully knocked down circ_0058063 expression (Fig. 2A). Then, the CDDP-resistant BC cells were subjected to CDDP treatment, and we used CCK-8 assay to detect the IC50 of T24/CDDP and 5637/CDDP cells. The IC50 value of sh-circ_0058063 was significantly lower than control in T24/CDDP and 5637/CDDP cells (Fig. 2B and C). Circ_0058063 silencing resulted in a significant downregulation of cell viability in T24/CDDP and 5637/CDDP cells co-treated with 6 μM CDDP (Fig. 2D and E). Moreover, we further estimated the effects of circ_0058063 knockdown on BC cell apoptosis. Apoptosis detection was shown in Fig. 2F and G, the apoptosis of T24/CDDP and 5637/CDDP cells in the sh-circ_0058063 group was significantly increased compared with the control group. In addition, to confirmed that circ_0058063 was associated with stem cell biomarkers, we analyzed SOX2, OCT4, and NANOG protein levels by western blot in T24/CDDP and 5637/CDDP cells. Interestingly, circ_0058063 knockdown significantly hampered expression levels of stem cell biomarkers in both T24/CDDP and 5637/CDDP cells (Fig. 2H-K). These findings support that circ_0058063 knockdown impeded the cell viability and induced apoptosis in CDDP-resistant BC cells.

### Circ_0058063 positively regulated B2M expression via sponging miR-335-5p

**Fig. 3:**
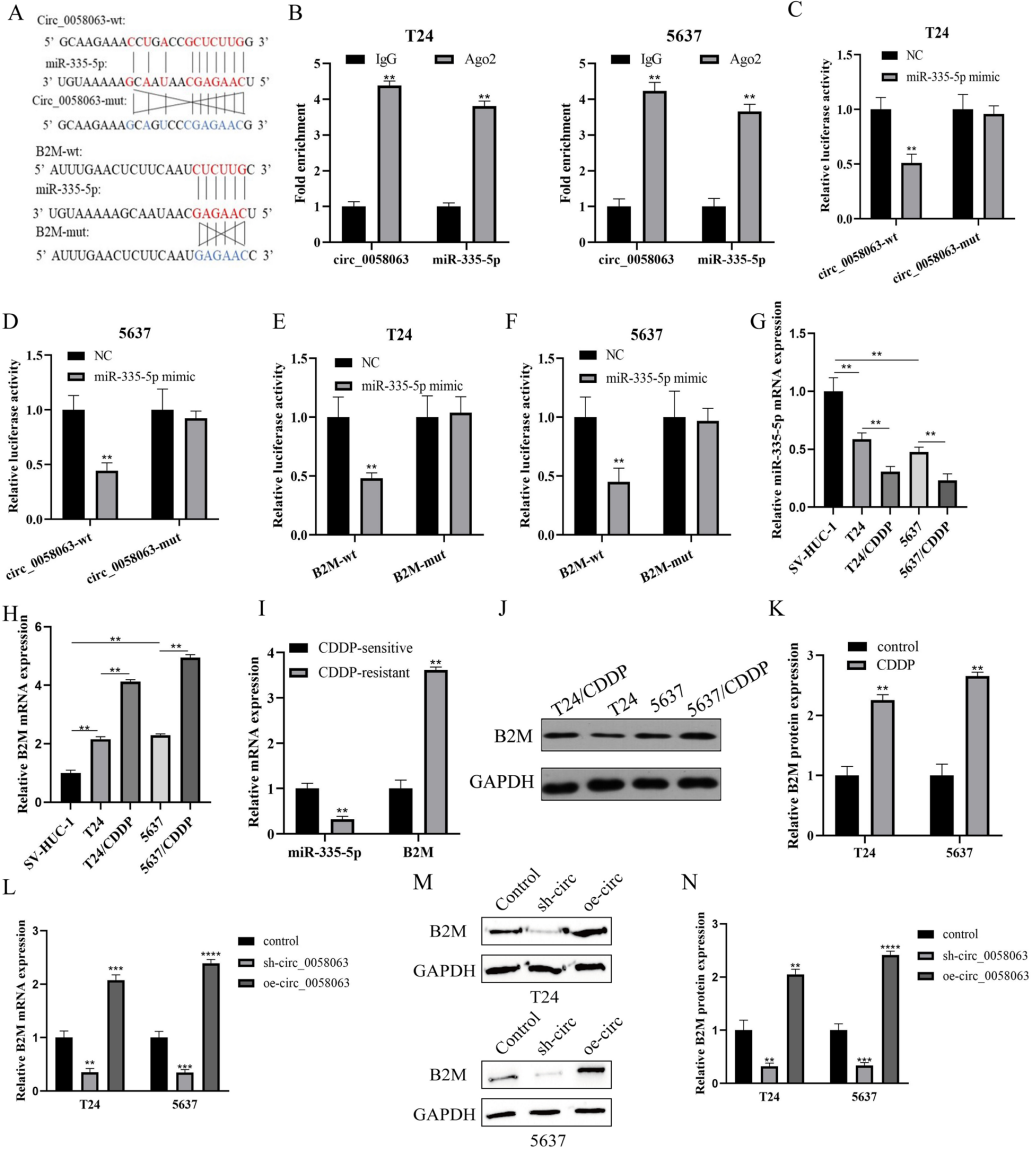
Circ_0058063 positively regulated B2M expression via sponging miR-335-5p. **A** The binding sites of circ_0085063/B2M (wild and mutant) and miR-335-5p were predicted by Starbase online database. **B** circ_0058063 and miR-335-5p co-immunoprecipitated with Ago2 revealed by RIP assay. **C**-**F** Dual-luciferase reporter assay examined the luciferase activity of wild/mutant circ_0058063 and B2M by miR-335-5p mimic or NC mimic. **G**, **H** The mRNA levels of miR-335-5p and B2M in SV-HVC-1, T24, T24/CDDP, 5637 and 5637/CDDP cell lines. **I** The mRNA levels of miR-335-5p and B2M in CDDP-resistant (*n* = 19) and CDDP-sensitive bladder cancer tissues (*n* = 16). **J**, **K** Western blot detected that the B2M protein expression was significantly increased in T-24/CDDP and 5637/CDDP cells compared with that in T24 and 5637 cells. **L** The B2M mRNA expression levels after knockdown or overexpression of circ_0058063 by RT-qPCR. **M**, **N** The B2M protein expression levels after knockdown or overexpression of circ-_0058063 by western blot. *P < 0.05

To further study the molecular mechanism of circ_0058063 and the effects on CDDP resistance, we predicted the possible binding site in miR-335-5p with circle RNA circ_0058063 and 3’ untranslated regions (3’-UTRs) of B2M mRNA by Starbase database (Fig. 3A). Firstly, RIP assay showed that the content of Ago2 binding to circ_0058063 and miR-335-5p binding was increased, as compared with IgG. This indicated that both circ_0058063 and miR-335-5p could bind to Ago2 protein (Fig. 3B). Furthermore, we mutated the binding sites of circ_0058063 and B2M for the next luciferase reporter assay. The results uncovered that the luciferase activity of wild-type circ_0058063 and B2M were decreased by mir-335-5p mimic, and the luciferase activity showed no apparent changes in cells with mutated circ_0058063 and B2M (Fig. 3C-F). Moreover, RT-qPCR assay revealed that miR-335-5p was decreased in T24 and 5637 cells, especially in T24/CDDP and 5637/CDDP cells (Fig. 3G). Conversely, B2M mRNA expression was increased especially in T24/CDDP and 5637/CDDP cells compared with T24 and 5637 cells (Fig. 3H). Besides, the levels of miR-335-5p and B2M mRNA were measured by RT-qPCR. MiR-335-5p was decreased and B2M was increased in CDDP-resistant tissues compared with CDDP-sensitive tissues (Fig. 3I). Meanwhile, western blot analysis also indicated that B2M expression was elevated in T24/CDDP and 5637/CDDP cells (Fig. 3J and K). Additionally, the mRNA and protein level of B2M was declined when circ_0058063 was downregulated, whereas overexpression of circ_0058063 had opposite effects on B2M protein in T24 and 5637 cells (Fig. 3L-N). Collectively, our results revealed that circ_0058063 regulated B2M expression by a sponging miR-335-5p.

### Circ_0058063 knockdown inhibited CDDP resistance in CDDP-resistant BC cells through targeting miR-335-5p

**Fig. 4:**
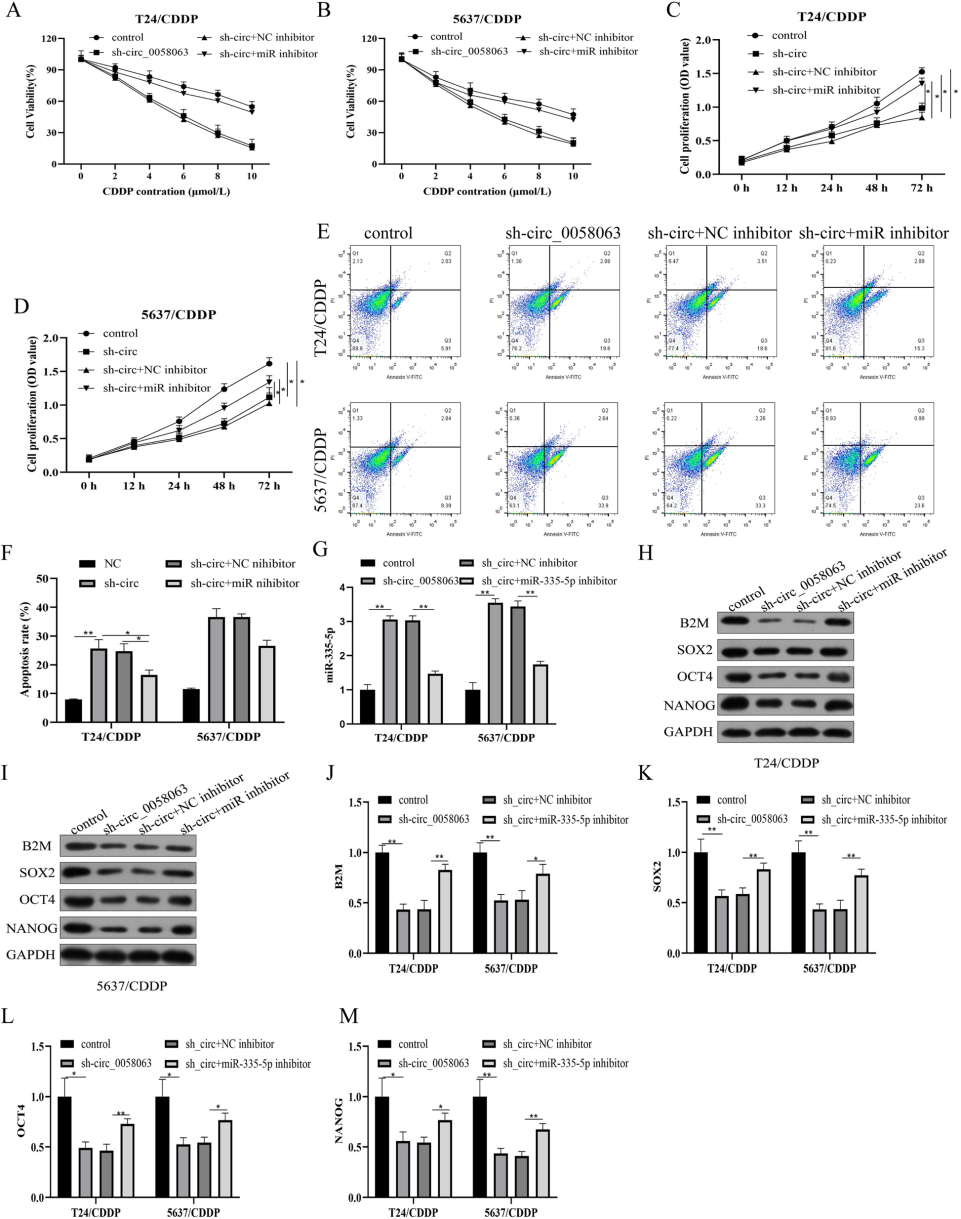
Circ_0058063 knockdown inhibited CDDP resistance in CDDP-resistant BC cells through targeting miR-335-5p. **A**, **B** The IC50 value of CDDP was measured by CCK-8 assay for 48 h. **C**, **D** Cell proliferation activity was estimated using CCK-8 assay. **E**, **F** The cell apoptosis rate was examined by Flow cytometry for 48 h. **G** The miR-335-5p level was measured by RT-qPCR. **H**-**M** The protein levels of B2M, SOX2, OCT4 and NANOG were detected by Western blot. *P < 0.05

Given that the circ_0058063/miR-335-5p/B2M axis had been identified in BC cells, we next confirmed that circ_0058063 regulated CDDP resistance of CDDP-resistant BC cells by sponging miR-335-5p. As shown in Fig. 4A and B, after CDDP treatment of CDDP-resistant cells, CCK-8 assay results disclosed that downregulation of miR-335-5p completely rescued the effect of knockdown circ_0058063 on IC50. The CCK-8 assay showed that miR-335-5p inhibitor partially rescued the cell viability in 6 μM CDDP-treated T24/CDDP and 5637/CDDP cells with circ_0058063 knockdown (Fig. 4C and D). Furthermore, the FCM was used to detect cell apoptosis, downregulation of circ_0058063 increased apoptosis ratio in T24/CDDP and 5637/CDDP cells, which were partially reversed by miR-335-5p inhibitor (Fig. 4E and F). In addition, miR-335-5p inhibitor partially decreased the effect of silencing circ_0058063 on miR-335-5p mRNA expression (Fig. 4G). Also, data in Fig. 4H-M indicated that the inhibiting effects of knockdown circ_0058063 on B2M and stem cell biomarkers were abrogated by miR-335-5p downregulation. All these results figured out that repression of miR-335-5p could reverse the effects of si-circ_0058063 on CDDP-resistant BC cell viability and stem cell biomarkers and apoptosis.

### Circ_0058063 ablation promoted cisplatin-sensitivity of BC cells *in vivo*

**Fig. 5:**
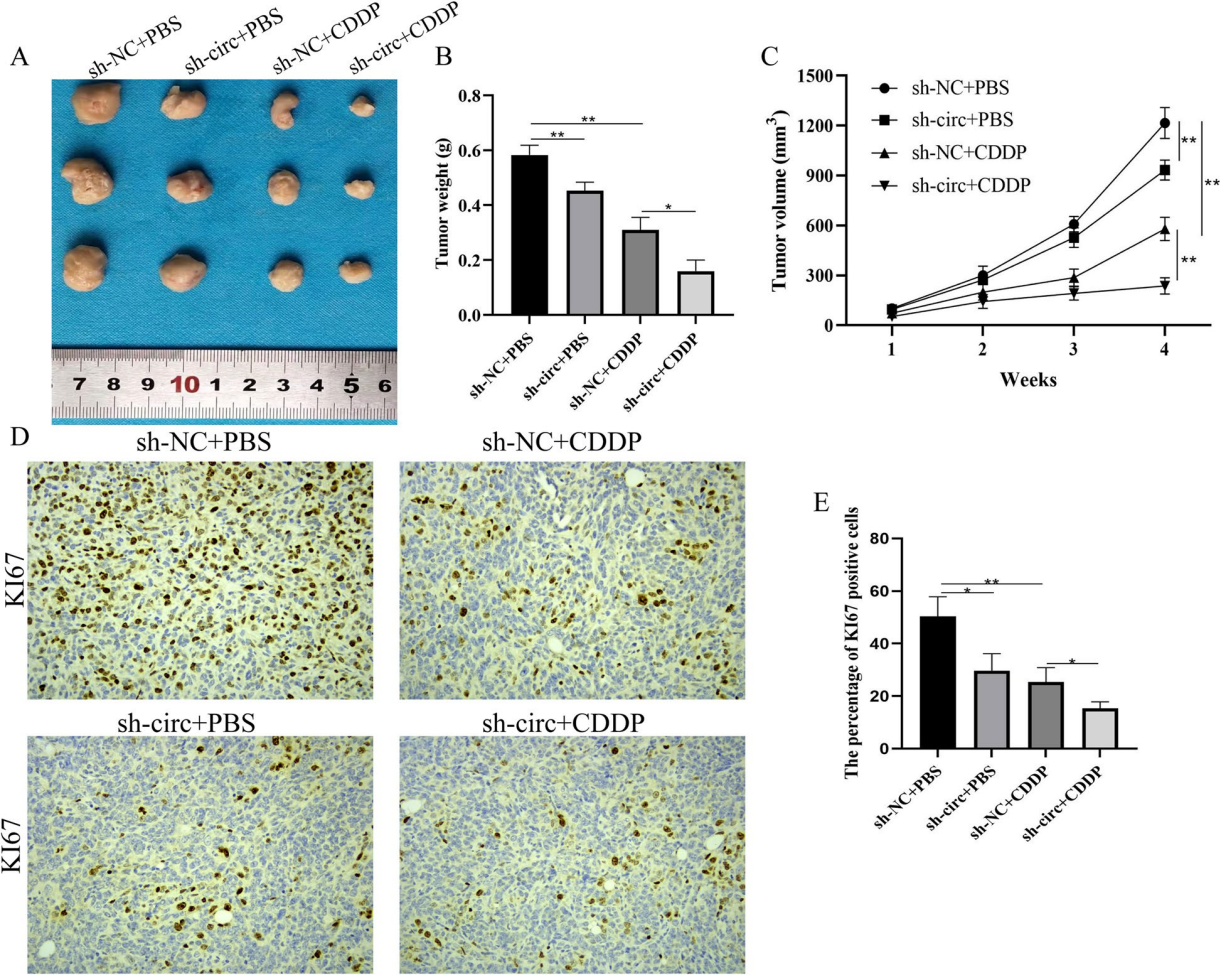
Circ_0058063 ablation promoted cisplatin-sensitivity of BC cells *in vivo*. **A** Xenograft tumors of mice with or without CDDP treatment (3 mg/kg, three times a week) at the end of the experiment. **B**, **C** Weight and Volume of tumors that developed in xenograft tumors. **D** The immunohistochemistry assay in xenografts from different groups. **E** The percentage of KI67 positive cells in xenografts from different groups. *P < 0.05

To investigate the effects of circ_0058063 on the chemosensitivity of BC cells to CDDP *in vivo*, T24/CDDP cells stably infected with sh-circ_0058063 or sh-NC were subcutaneously injected into mice, followed by administration with CDDP or PBS. As expected, circ_0058063 downregulation inhibited BC tumor growth in CDDP-resistant cells (Fig. 5A). Tumor weight and volume were lower in sh-circ_0058063 + CDDP group than sh-NC + CDDP group than sh- circ_0058063 + PBS group than sh-NC + PBS group (Fig. 5B and C). Consistently, the immunohistochemistry assay revealed that when circ_0058063 was knockdown, KI67 in sh-circ_0058063 + CDDP group were significantly suppressed (Fig. 5D and E). Taken together, knockdown of circ_0058063 exerted suppressive effects on tumor growth of CDDP-resistant BC cells *in vivo*.

## Discussion

BC is the most common genitourinary system malignant tumor [28], and CDDP is one of the commonly used drugs for chemotherapy of BC [29]. However, drug resistance is the main reason for the failure of BC treatment [30]. Therefore, identification of the promising targets that enhance CDDP-sensitivity may help to increase the therapeutic efficacy of this chemical drug in clinical practices. Among all the cancer-associated genes, more and more evidences show that circular RNAs play a key role in regulating chemo-resistance of various types of malignant tumors. For example, circ_0006528 is overexpressed in paclitaxel-resistant breast cancer tissues and cells [20]. Also, circular RNA circ_002639 was increased in CDDP-resistance of gastric cancer and was associated with poor survival rates [31]. Circ_0058063 has been reported as an oncogene to facilitate the progression of BC [14, 15], but its role in regulating CDDP-resistance has not been explored. In our study, we verified that circ_0058063 expression levels were increased in CDDP-resistance BC tissues and cell lines and was associated with poor prognosis, and silencing of circ_0058063 suppressed proliferation and stem cell characteristics and promotes apoptosis of T24/CDDP and 5637/CDDP cells, indicating that circ_0058063 ablation enhanced CDDP-sensitivity in BC.

Many studies have indicated that circRNAs can sponge miRNAs to positively regulate the mRNA levels of the downstream targets through targeting their 3’ untranslated regions (3’-UTR), which is known as the competing endogenous RNA (ceRNA) mechanisms [32]. For example, hsa_circ_0091570 serves as a ceRNA sponge for miR-1307 to upregulate ISM1 expression and promotes the progression of hepatocellular cancer [33], and hsa_circ_0074834 boosts osteogenic differentiation of bone nonunion mesenchymal stem cells and bone defect repair through miR-942-5p regulate the expression of VEGF and ZEB1 [34]. In consistent with the above literatures, our research evidenced that circ_0058063 sponged miR-335-5p to induce B2M upregulation in a ceRNA-dependent manner. Besides, the circRNAs/miRNAs axis has been widely been reported to regulate chemo-resistance, for example, Sang et al. report that circRNA_0025202 suppressed tumor progression and sensitivity to tamoxifen through the miR-182-5p/FOXO3a axis in breast cancer [35], and Huang et al. indicate that circAKT3 promotes PI3KR1 expression by sponging miR-198 to elevate the CDDP-resistant properties in gastric cancer [36]. In this study, we verified that circ_0058063 also regulated CDDP-resistance in BC through targeting miR-335-5p. Specifically, we found that the promoting effects of circ_0058063 on CDDP-sensitivity in BC cells were reversed by suppressing miR-335-5p. In addition, Beta-2-microglobulin (B2M) is an endogenous low molecular weight serum protein that plays an important role in the immune response and angiogenesis. Research has shown that B2M was high expression in prostate cancer cells and increased B2M was related to distance metastasis [37], and increased serum levels of B2M and FLT3-L in patients with multiple myeloma may be associated with increased angiogenesis and myelosuppression [38]. In our findings, miR-335-5p was able to bind the 3’-UTR of B2M, and we found that knockdown circ_0058063 down-regulated the expression of B2M by sponging miR-335-5p, suggesting that the circ_0058063/miR-335-5p axis might exert their regulating effects on CDDP-resistance in BC through targeting B2M. However, the detailed molecular mechanisms were still needed to be explored in our future work.

Cancer stem cells (CSCs) play a crucial role in drug resistance of many cancers [39–41]. Mechanistically, during cancer aggressiveness, the cancer cells show heterogenous characteristics in tumors, which contain both undifferentiated CSCs and the differentiated cancer cells. Under chemical stresses, the CSCs will differentiate into the cancer cells with drug-resistant properties, and expansion of this subgroup of cells will make cancer more resistant to chemical drug. Thus, elimination of CSCs is a novel strategy to improve chemo-sensitivity [39–41]. In recent studies, various cancer associated genes were identified to be closely associated with chemo-resistance, for example, overexpression of miR-124 repressed liver CSCs and sorafenib resistance [42], lncRNA NEAT1 contributed to CSC-like properties in CDDP-resistance non-small cell lung cancer cells [43], and MYPT1 downregulation increases platinum resistance in ovarian cancer by increasing the stemness [44]. Similar to the above publications, in this study, we disclosed that knockdown of circ_0058063 suppressed CSCs generation in the CDDP-resistant BC cell through sponging miR-335-5p, suggesting that targeting the circ_0058063/miR-335-5p axis is effective to restrain CSCs generation in CDDP-resistant BC cells. The above results were partially supported by the previous study that miR-335-5p negatively regulates CSC properties in osteosarcoma [45]. Overall, these results indicated that circ_0058063 ablation was effective to suppress stem cell biomarkers and increase CDDP-sensitivity in BC cells.

## Conclusions

In conclusion, this study found that knockdown of circ_0058063 increased CDDP-sensitivity and suppressed stem cell biomarkers in BC, and B2M might be the downstream effector gene, and we provided evidences that the circ_0058063/miR-355-5p/B2M axis may serve as the foundation for the development of novel potential therapeutic strategies to enhance CDDP-sensitivity in BC.

## Supplementary Information

**Additional file 1.** 

## Data Availability

All data generated or analyzed during this study are included in this published article (and its supplementary information files).

## References

[1] Borden LSJ, Clark PE, Hall MC (2005). Bladder cancer. Curr Opin Oncol.

[2] Antoni S, Ferlay J, Soerjomataram I, Znaor A, Bray FJEU (2016). Bladder cancer incidence and mortality: a global overview and recent trends. Eur Urol.

[3] Yi L, Kaisu L, Zhao Y, Ning H, Xiaofeng Q, Xiaoyang G, Chong L (2017). Bladder cancer stem cells: clonal origin and therapeutic perspectives. Oncotarget.

[4] Liu P, Li X, Cui Y, Chen J, Li C, Li Q, Li H, Zhang X, Zu X (2019). LncRNA-MALAT1 mediates cisplatin resistance via miR-101-3p/VEGF-C pathway in bladder cancer. Acta Biochim Biophys Sin (Shanghai).

[5] Jin J, Sun H, Shi C, Yang H, Wu Y, Li W, Dong YH, Cai L, Meng XM (2020). Circular RNA in renal diseases. J Cell Mol Med.

[6] M-Ashraf A, Tiffany N, Afaan K, Kexiang L, Xiufen Z. Circular RNA in cardiovascular disease. J Cell Physiol. 2018;234(5):5588–600. 10.1002/jcp.27384 30341894

[7] Wang Y, Liu B (2020). Circular RNA in diseased heart. Cells.

[8] Chen D, Ma W, Ke Z, Xie F (2018). CircRNA hsa_circ_100395 regulates miR-1228/TCF21 pathway to inhibit lung cancer progression. Cell Cycle.

[9] Bi J, Liu H, Cai Z, Dong W, jiang N, Yang M, Huang J, Lin T (2018). Circ-BPTF promotes bladder cancer progression and recurrence through the miR-31-5p RAB27A axis. Aging.

[10] Li T, Sun X, Chen L (2019). Exosome circ_0044516 promotes prostate cancer cell proliferation and metastasis as a potential biomarker. J Cell Biochem.

[11] Zhang W, Wang Z, Cai G, Huang P (2021). Downregulation of Circ_0071589 suppresses cisplatin resistance in colorectal cancer by regulating the MiR-526b-3p/KLF12 Axis. Cancer Manag Res.

[12] Liu S, Wu M, Peng M (2020). Circ_0000260 Regulates the Development and deterioration of gastric adenocarcinoma with cisplatin resistance by upregulating MMP11 via targeting MiR-129-5p. Cancer Manag Res.

[13] Li X, Ding L, Gu G, Zheng C, Pan C, Zheng Q, Xiang T (2021). Role and mechanism of circ_0058063/miR-635 axis in the malignant phenotype of multiple myeloma RPMI8226 cells. Evid Based Complement Alternat Med.

[14] Liang H, Huang H, Li Y, Lu Y, Ye T (2020). CircRNA_0058063 functions as a ceRNA in bladder cancer progression via targeting miR-486–3p/FOXP4 axis. Biosci Rep.

[15] Sun M, Zhao W, Chen Z, Li M, Li S, Wu B, Bu R (2019). Circ_0058063 regulates CDK6 to promote bladder cancer progression by sponging miR-145-5p. J Cell Physiol.

[16] Zheng Y, Chen Y, Jiang H, Zhang H, Wang H, Xu J, Yu Z (2020). Circ_0058063 upregulates GLUT1 expression and promotes glucose-uptake in esophageal squamous-cell carcinomas. J Thorac Dis.

[17] Kulcheski FR, Christoff AP, Margis RJ (2016). Circular RNAs are miRNA sponges and can be used as a new class of biomarker. J Biotechnol.

[18] Zhang S, Cheng J, Quan C, Wen H, Feng Z, Hu Q, Zhu J, Huang Y, Wu X (2020). circCELSR1 (hsa_circ_0063809) contributes to paclitaxel resistance of ovarian cancer cells by regulating FOXR2 expression via miR-1252. Mol Ther Nucleic Acids.

[19] Huang X, Li Z, Zhang Q, Wang W, Li B, Wang L, Xu Z, Zeng A, Zhang X, Zhang X, He Z, Li Q, Sun G, Wang S, Li Q, Wang L, Zhang L, Xu H, Xu Z (2019). Circular RNA AKT3 upregulates PIK3R1 to enhance cisplatin resistance in gastric cancer via miR-198 suppression. Mol Cancer.

[20] Liu G, Zhang Z, Song Q, Guo Y, Bao P, Shui H (2020). Circ_0006528 contributes to paclitaxel resistance of breast cancer cells by regulating miR-1299/CDK8 axis. Onco Targets Ther.

[21] Wang X, Xiao H, Wu D, Zhang D, Zhang Z (2020). miR-335-5p regulates cell cycle and metastasis in lung adenocarcinoma by targeting CCNB2. Onco Targets Ther.

[22] Yong W, Zeng X, Wang N, Wei Z, Zhang X, Teng S, Zhang Y, Lu Z (2018). Long noncoding RNA DANCR, working as a competitive endogenous RNA, promotes ROCK1-mediated proliferation and metastasis via decoying of miR-335-5p and miR-1972 in osteosarcoma. Mol Cancer.

[23] Zhang D, Yang N (2019). MiR-335-5p inhibits cell proliferation, migration and invasion in colorectal cancer through downregulating LDHB. J BUON.

[24] Liu R, Guo H, Lu S (2018). MiR5 restores cisplatin sensitivity in ovarian cancer cells through targeting BCL2L2. Cancer Med.

[25] Bagrodia A, Lee BH, Lee W, Cha EK, Sfakianos JP, Iyer G, Pietzak EJ, Gao SP, Zabor EC, Ostrovnaya I (2016). Genetic determinants of cisplatin resistance in patients with advanced germ cell tumors. J Clin Oncol.

[26] Tanaka N, Miyajima A, Kosaka T, Shirotake S, Hasegawa M, Kikuchi E, Oya M (2010). Cis-dichlorodiammineplatinum upregulates angiotensin II type 1 receptors through reactive oxygen species generation and enhances VEGF production in bladder cancer. Mol Cancer Ther.

[27] Wang X, Xiao H, Wu D, Zhang D, Zhang ZJO (2020). miR-335-5p regulates cell cycle and metastasis in lung adenocarcinoma by targeting CCNB2. Onco Targets Ther.

[28] Seidl C (2020). Targets for therapy of bladder cancer. Semin Nucl Med.

[29] Godwin JL, Hoffman-Censits J, Plimack E (2018). Recent developments in the treatment of advanced bladder cancer. Urol Oncol.

[30] Roh YG, Mun MH, Jeong MS, Kim WT, Leem SH (2018). Drug resistance of bladder cancer cells through activation of ABCG2 by FOXM1. BMB Rep.

[31] Zhang Z, Yu X, Zhou B, Zhang J, Chang J (2020). Circular RNA circ_0026359 enhances cisplatin resistance in gastric cancer via targeting miR-1200/POLD4 pathway. Biomed Res Int.

[32] Li X, Ding J, Wang X, Cheng Z, Zhu Q (2020). NUDT21 regulates circRNA cyclization and ceRNA crosstalk in hepatocellular carcinoma. Oncogene.

[33] Wang YG, Wang T, Ding M, Xiang SH, Zhai B (2019). hsa_circ_0091570 acts as a ceRNA to suppress hepatocellular cancer progression by sponging hsa-miR-1307. Cancer Lett.

[34] Ouyang Z, Tan T, Zhang X, Wan J, Zhou Y, Jiang G, Yang D, Guo X, Liu T (2019). CircRNA hsa_circ_0074834 promotes the osteogenesis-angiogenesis coupling process in bone mesenchymal stem cells (BMSCs) by acting as a ceRNA for miR-942-5p. Cell Death Dis.

[35] Sang Y, Chen B, Song X, Li Y, Liang Y, Han D, Zhang N, Zhang H, Liu Y, Chen T, Li C, Wang L, Zhao W, Yang Q (2019). circRNA_0025202 regulates tamoxifen sensitivity and tumor progression via regulating the miR-182-5p/FOXO3a axis in breast cancer. Mol Ther.

[36] Huang X, Li Z, Zhang Q, Wang W, Li B, Wang L, Xu Z, Zeng A, Zhang X, Zhang X, He Z, Li Q, Sun G, Wang S, Li Q, Wang L, Zhang L, Xu H, Xu Z (2019). Circular RNA AKT3 upregulates PIK3R1 to enhance cisplatin resistance in gastric cancer via miR-198 suppression. Mol Cancer.

[37] Abdul M, Hoosein N (2000). Changes in beta-2 microglobulin expression in prostate cancer. Urol Oncol.

[38] Kokonozaki M, Kanellou P, Pappa CA, Vyzoukaki R, Sarantoulaki S, Stavroulaki E, Kyriakaki S, Alegakis A, Boula A, Alexandrakis MG (2017). Serum levels of soluble FLT3 ligand in patients with active multiple myeloma constitute marker of bone marrow plasma cell proliferative activity. Crit Rev Oncog.

[39] Carotenuto P, Hedayat S, Fassan M, Cardinale V, Lampis A, Guzzardo V, Vicentini C, Scarpa A, Cascione L, Costantini D, Carpino G, Alvaro D, Ghidini M, Trevisani F, Te Poele R, Salati M, Ventura S, Vlachogiannis G, Hahne JC, Boulter L, Forbes SJ, Guest RV, Cillo U, Said-Huntingford I, Begum R, Smyth E, Michalarea V, Cunningham D, Rimassa L, Santoro A, Roncalli M, Kirkin V, Clarke P, Workman P, Valeri N, Braconi C (2020). Modulation of biliary cancer chemo-resistance through MicroRNA-mediated rewiring of the expansion of CD133+ cells. Hepatology.

[40] El-Sahli S, Wang L (2020). Cancer stem cell-associated pathways in the metabolic reprogramming of breast cancer. Int J Mol Sci.

[41] Wu H, Liu B, Chen Z, Li G, Zhang Z (2020). MSC-induced lncRNA HCP5 drove fatty acid oxidation through miR-3619-5p/AMPK/PGC1α/CEBPB axis to promote stemness and chemo-resistance of gastric cancer. Cell Death Dis.

[42] Feng Y, Jiang W, Zhao W, Lu Z, Dong Y (2020). miR-124 regulates liver cancer stem cells expansion and sorafenib resistance. Exp Cell Res.

[43] Jiang P, Xu H, Xu C, Chen A, Chen L, Zhou M, Haq IU, Wu X, Mariyam Z, Feng Q (2018). NEAT1 contributes to the CSC-like traits of A549/CDDP cells via activating Wnt signaling pathway. Chem Biol Interact.

[44] Muoz-Galván S, Felipe-Abrio B, Verdugo-Sivianes EM, Perez M, Carnero A (2020). Downregulation of MYPT1 increases tumor resistance in ovarian cancer by targeting the Hippo pathway and increasing the stemness. Mol Cancer.

[45] Guo X, Yu L, Zhang Z, Dai G, Gao T, Guo W (2017). miR-335 negatively regulates osteosarcoma stem cell-like properties by targeting POU5F1. Cancer Cell Int.

