# Supplementary material for: Circ_0058063 contributes to cisplatin-resistance of bladder cancer cells by upregulating B2M through acting as RNA sponges for miR-335-5p
Source: BMC Cancer. 2022 Mar 23;22:313. doi: 10.1186/s12885-022-09419-1 (PMC8943922; doi:10.1186/s12885-022-09419-1)

Western blot

Figure 2H SOX2 in T24/CDDP cells

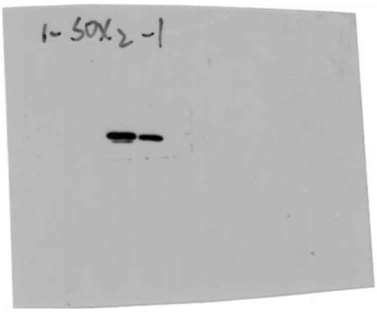

Figure 2H OCT4 in T24/CDDP cells

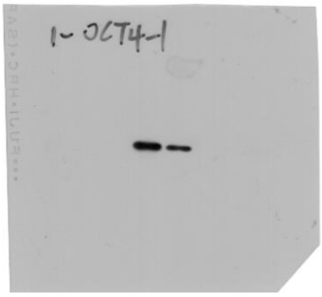

Figure 2H NANOG in T24/CDDP cells

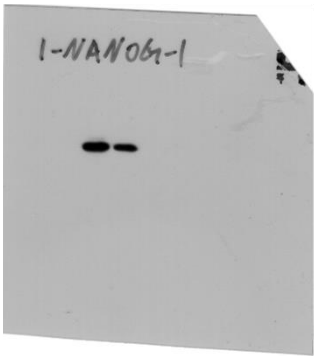

Figure 2H GAPDH in T24/CDDP cells

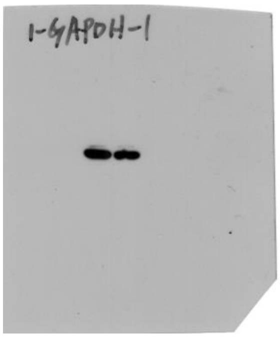

Figure 2I SOX2 in T24/CDDP cells

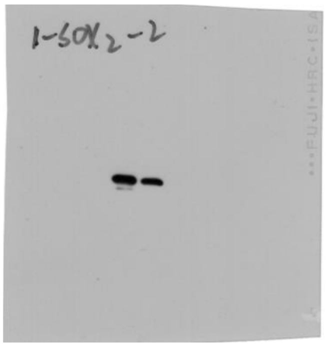

Figure 2I OCT4 in T24/CDDP cells

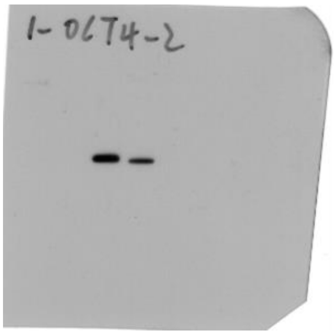

Figure 2I NANOG in T24/CDDP cells

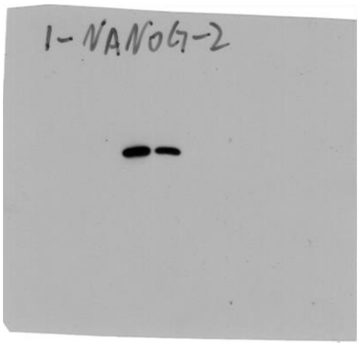

Figure 2I GAPDH in T24/CDDP cells

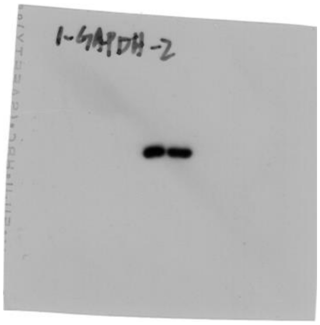

Figure 3J B2M

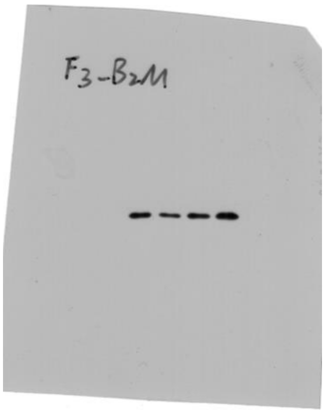

Figure 3J GAPDH

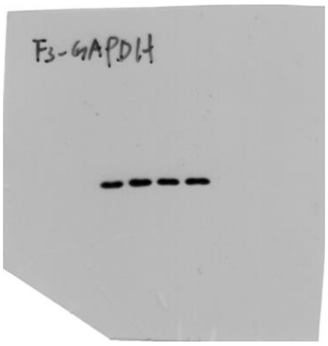

Figure 4H B2M

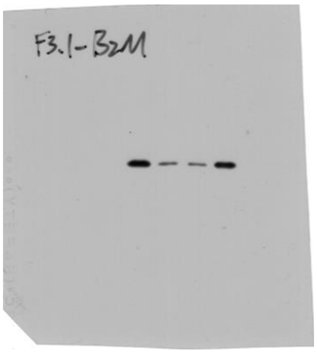

Figure 4H SOX2

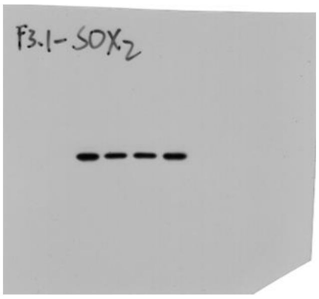

Figure 4H OCT4

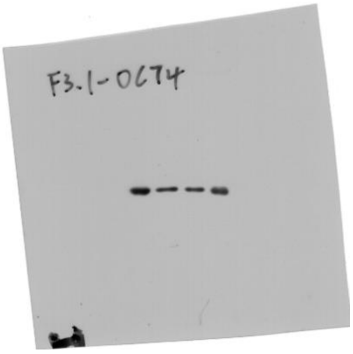

Figure 4H NANOG

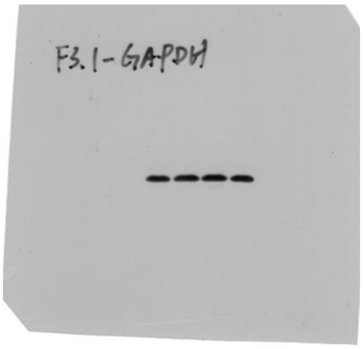

Figure 4I B2M

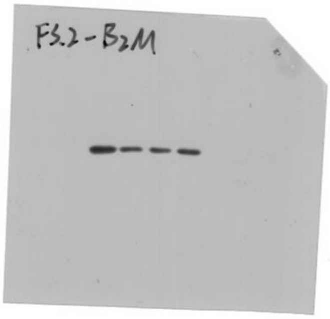

Figure 4I SOX2

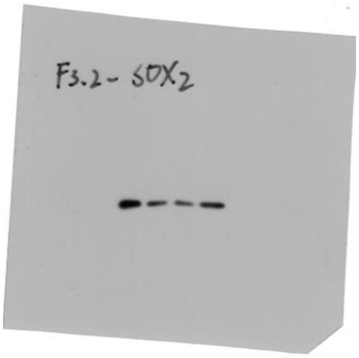

Figure 4I OCT4

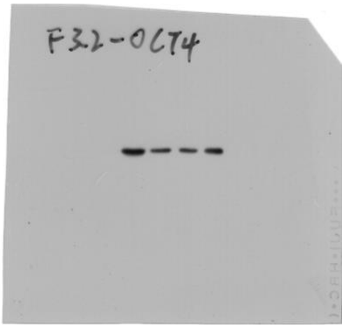

Figure 4I NANOG

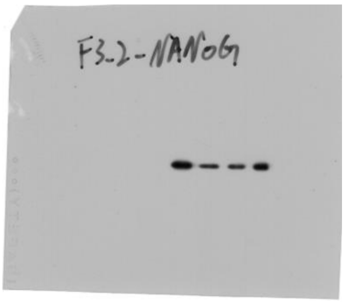

Figure 4I GAPDH

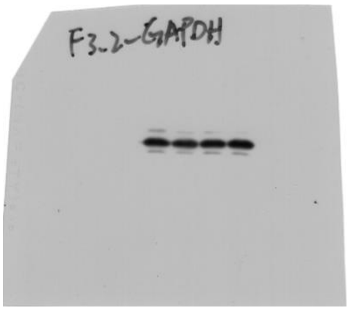

Supplement

Figure 3M B2M in T24 cells

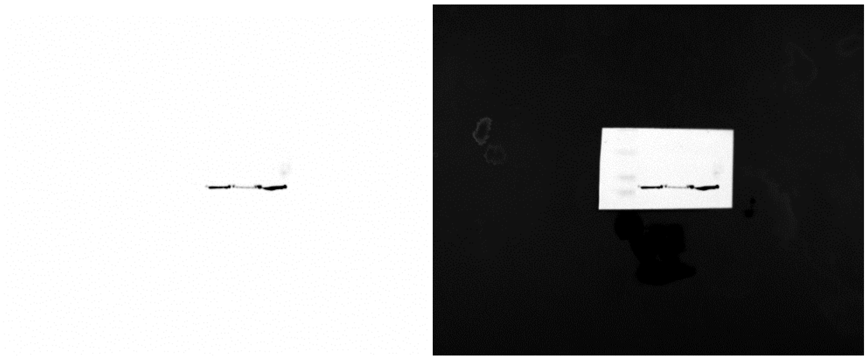

Figure 3M GAPDH in T24 cells

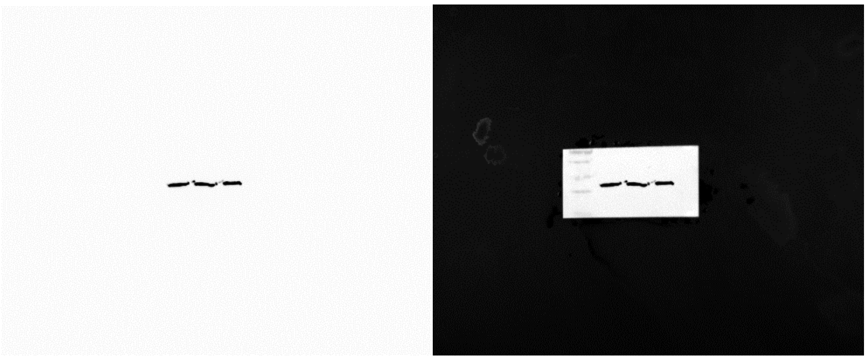

Figure 3M B2M in 5637 cells

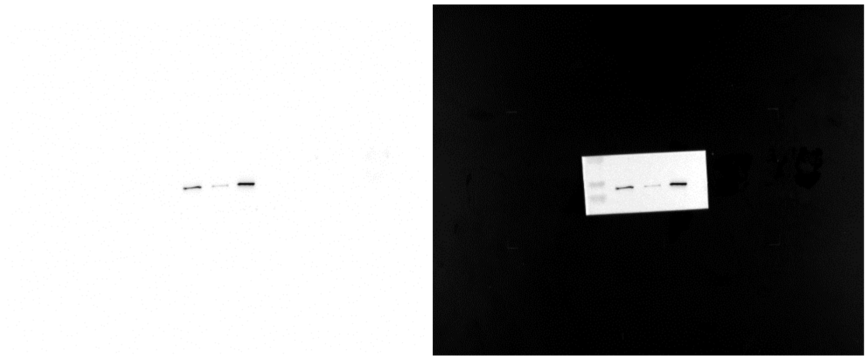

Figure 3M GAPDH in 5637 cells

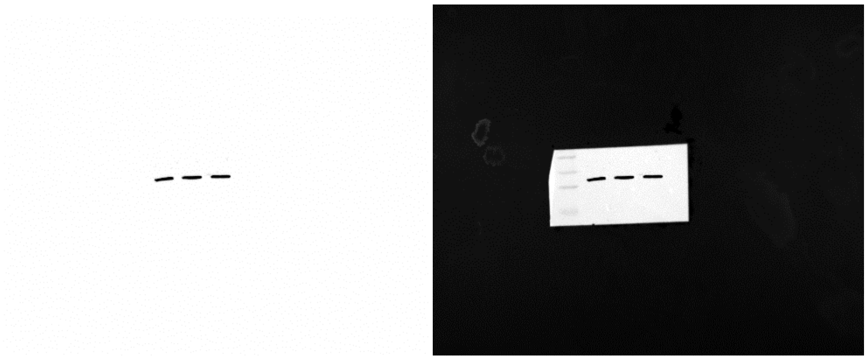

Supplement: Supplementary file 1 — Additional file 1. [file 12885_2022_9419_MOESM1_ESM.pdf]
